# Investigating Anisotropic Magnetoresistance in Epitaxially Strained CoFe Thin Films on a Flexible Mica

**DOI:** 10.3390/nano13243154

**Published:** 2023-12-16

**Authors:** Guang-Yang Su, Min-Chang You, Kai-Wei Chuang, Ming-Hsuan Wu, Cheng-Hsun Hsieh, Chun-Yen Lin, Chao-Yao Yang, Aswin kumar Anbalagan, Chih-Hao Lee

**Affiliations:** 1 Department of Engineering and System Science, National Tsing Hua University, Hsinchu 30013, Taiwan; watch159159159@gmail.com (G.-Y.S.); ghkl22025@gmail.com (M.-C.Y.); m0952689709@gmail.com (M.-H.W.); 2 Institute of Nuclear Engineering and Science, National Tsing Hua University, Hsinchu 30013, Taiwan; jk733270107@gmail.com; 3 Department of Materials Science and Engineering, National Yang Ming Chiao Tung University, Hsinchu 30013, Taiwan; nicky100585@gmail.com (C.-H.H.); cyyang8611@nycu.edu.tw (C.-Y.Y.); 4 National Synchrotron Radiation Research Center, Hsinchu 30076, Taiwan; lin42077@gmail.com

**Keywords:** CoFe films, epitaxial growth, VSM, flexible substrate, AMR sensor

## Abstract

This study investigates the crystal structure, epitaxial relation, and magnetic properties in CoFe thin films deposited on a flexible mica substrate. The epitaxial growth of CoFe thin films was successfully achieved by DC magnetron sputtering, forming three CoFe(002) domains exhibiting four-fold symmetry on the mica substrate. A notable achievement of this work was the attainment of the highest anisotropic magnetoresistance (AMR) value reported to date on a flexible substrate. Additionally, it was observed that the magnetic characteristics of the CoFe films on the flexible mica substrate display reversibility upon strain release. More importantly, the AMR effect of epitaxial CoFe films on flexible mica shows lesser dependence on the crystalline orientation and remains the same under different bending states. These findings demonstrate the potential of utilizing CoFe films on flexible substrates to develop wearable magnetoresistance sensors with diverse applications.

## 1. Introduction

Recently the demand for flexible sensors has increased across diverse sectors including electronics and health monitoring systems [1]. Organic polymer materials have merged as suitable choices due to their properties such as flexibility, ductility, and lightweight nature [2]. This kind of flexible sensor can attain perfect contact on curved or human surfaces, thereby making miniaturization and integration possible [3].

With the development of flexible electronics, flexible substrates have attracted potential applications such as wearable devices, piezoelectric sensors, etc., due to their higher sensitivity and deformation stability [4,5,6]. Over the last few decades, various magnetic systems have focused on utilizing flexible substrates for spintronic and magneto-electronic applications [7,8]. In general, the magnetic properties of the thin films depend on the strain induced either internally or externally. There are various methods to induce stress in magnetic thin films: strain resulting from the lattice mismatch between the thin film and substrate [9], the growth of magnetic films on a piezoelectric substrate, along with the application of an electric field [10,11], and the growth of magnetic films on a flexible substrate, which involves the application of mechanical force based on the Villari effect [12,13,14].

The Villari effect describes the phenomenon where external stress is induced, subsequently influencing the magnetic properties of the material. The magnetostrictive constant (λ_ms_) is defined as λ_ms_ = Δl/l, where Δl signifies the change in length along the direction of the applied magnetic field and l represents the original length of the material. This dimensionless ratio represents the material’s elongation or contraction concerning its original length after exposure to an external magnetic field. Materials with a positive magnetostrictive constant exhibit elongation along the magnetic field direction when subjected to an external magnetic field. Similarly, when external forces induce tensile strain, these materials exhibit a larger magnetization along the stretched direction compared to their unstretched state. This behavior highlights the intricate relationship between magnetic fields, material dimensions, and resulting magnetic properties in materials with positive magnetostrictive constants. However, most flexible devices built on polymer-based substrates cannot tolerate higher temperatures. Therefore, alternative flexible substrates with higher melting points and comparable performance to rigid substrates are crucial.

In this regard, muscovite (mica), a ceramic mineral, has become a popular material for flexible substrates due to its high melting point, excellent transparency, flexibility, and outstanding chemical stability. Its layered structure enables epitaxial film growth with minimal imposition of stress due to weak van der Waals forces instead of stronger covalent or ionic bonds. Therefore, the mica substrate does not significantly distort the lattice of the epitaxial film. This results in van der Waals heteroepitaxy, where the lattice constant of the epitaxial film tends to be closer to that of the bulk material. Additionally, despite a lattice mismatch of up to approximately 58%, epitaxy can still occur, and defects resulting from this lattice mismatch are minimized due to the relatively weak van der Waals forces between the film and mica [15,16,17].

Recently, the growth of ferromagnetic thin films on flexible substrates has emerged as a significant research field, as these structures are considered ideal for application in flexible magnetic field sensors based on the anisotropic magnetoresistance (AMR) effect. The AMR ratio is expressed by Equation (1) as shown below.

(1) $$AMR\%=\left(R_{∥}-R_{⊥}\right)/R_{⊥}$$

where the $R_{∥}$ and $R_{⊥}$ in Equation (1) are denoted as the resistance states have taken while the magnetization of the AMR sensor is parallel and transverse to the sensing current, respectively. This kind of reversal process between these low-resistance and high-resistance states results in a linear output as a function of an external applied magnetic field. In comparison with conventional Hall sensors, AMR sensors have tremendous potential for better sensitivity and integrated magnetic sensing, which is widely employed in magnetic signal detections such as magnetic card readers, magnetic switch, etc. [18,19].

Although the amplitudes of the AMR ratio are much smaller compared to the counterparts of giant magnetoresistance (GMR) and tunneling magnetoresistance (TMR), the basic architecture of an AMR sensor only requires a single magnetic layer, eliminating the need for the complicated spin-valve structure found in GMR and TMR. This design of AMR significantly simplifies the stacking structure, avoiding issues arising from the heterostructure interfaces. Additionally, it is important to note that using a flexible substrate for AMR sensors can offer numerous application possibilities due to demonstrated improvement in AMR ratios and excellent deformation stability in several systems [20,21,22]. A recent work conducted by Wang et al. showed that the NiFe thin film on a PET substrate yielded an AMR ratio of 0.96% [20]. In addition, Chen et al. investigated the AMR sensor based on a NiFe film on a Kapton substrate yielding a stable AMR ratio of approximately 1% at different bending curvatures [21]. These works discuss the utilization of NiFe-based systems for AMR sensors; however, no work on CoFe-based systems for AMR sensors has been reported so far.

CoFe films are a popular material in spintronic devices, especially in Magnetoresistive Random Access Memory (MRAM). Their advantages, such as low energy use, quick access and writing, non-volatility, and long lifespan, make them fascinating for many research teams. CoFe films are still a subject of interest due to their great performance. In MRAM, their popularity continues as scientists explore their potential in various applications. Researchers are eager to uncover more about CoFe films and their role in advancing spintronic technologies.

In addition to MRAM, CoFe is considered a promising spintronic candidate for magnetic sensors due to its remarkable properties such as large saturation magnetization, high Curie temperature, and high permeability [23]. Parkin et al. reported an epitaxially coherent relationship between MgO and CoFe layers, resulting in an atomically flat interface when they are combined [24]. Moreover, the AMR effect in epitaxially grown ferromagnet is strongly influenced by crystal symmetry. Therefore, it is undeniable that the precise control over the crystal structure of the ferromagnet plays a crucial role in the performance of the CoFe-based AMR devices, which have been extensively studied [25,26,27,28,29]. However, these studies have demonstrated only a static aspect of the CoFe-based AMR devices, as the magnetic properties of CoFe were fixed once the structures were completed. Therefore, thanks to the additional structural degree of freedom resulting from the strain/stress issues arising from the flexible substrate, integrating CoFe with a flexible substrate could introduce another dynamic dimension to AMR devices. These flexible AMR devices show considerable potential for wearable sensors compared to GMR or TMR, which is primarily due to their greater strain compatibility. Compared to a polymer substrate, mica is prominent for its environment-friendly nature, higher bendability, excellent transparency, and higher melting point [30].

This work presents an investigation into the microstructural and magnetic properties of a CoFe thin film epitaxially grown on a mica substrate, as well as with the associated AMR performance under various applied strain conditions. While the utilization of mica as a flexible substrate holds potential advantages in many aspects, the application of CoFe/mica-based systems for AMR devices has not yet been studied. Consequently, this study primarily focuses on establishing a triangular correlation among microstructure, magnetism, and the transport properties in the CoFe/mica, with a specific emphasis on observing stability changes under applied strain. For magnetic sensor applications in flexible devices, it is of vital importance to minimize the impact of stress or strain effects. In this work, we applied stress/strain on CoFe films and found that the AMR effect was not significantly affected by the applied stress/strain on the CoFe thin film sample.

## 2. Experimental Section

CoFe thin films were prepared using DC magnetron sputtering from a 2-inch^2^ Co_50_Fe_50_ alloy target with a purity of 99.99%. CoFe thin films (50 nm) were deposited on mica substrates at 400 °C to observe the effect of growth temperature during deposition. The base pressure of the sputtering chamber was maintained at around 2 × 10^−6^ Torr and the sputtering was carried out at 15 W power in an argon atmosphere at a working pressure of about 5 × 10^−3^ Torr.

Synchrotron-based X-ray diffraction (XRD) measurements were performed at BL 17 B of the National Synchrotron Radiation Research Center (NSRRC), Taiwan, to investigate the microstructural properties such as crystallinity, growth crystallography, and the epitaxial relationship in CoFe/mica samples. The beamline end station was equipped with a six-circle Huber diffractometer, which is a combination of standard Eulerian geometry Huber 5020 on two circle rotation tables used for adjusting grazing incidence angles in the in-plane X-ray diffraction experiments. The thickness of the CoFe thin films was measured using the X-ray reflectivity (XRR) technique. XRR measurements were performed using a lab-based X-ray source emitting Cu K_α_ radiation (PANalytical X’Pert Pro, Westborough, MA, USA, λ = 1.54056 Å). The magnetization measurements were performed using vibrating sample magnetometry (VSM, Lake Shore Cryotronics PMC-3900, Westerville, OH, USA). To study the strain-induced magnetization on CoFe thin films, mica substrates were exfoliated to 50 μm in thickness. Samples were mounted to distinct bending molds made from polyethylene and shaped using a 3D printer to facilitate various strain conditions, thereby enabling corresponding magnetization measurements for each strain condition. For the AMR measurements, Au electrodes were deposited on the CoFe thin films and a current of 100 μA was applied in the CoFe thin film. The AMR response was measured using a Keithley 2400 source meter (Cleveland, OH, USA) with a two-probe method at an applied field of 2500 Oe.

## 3. Results and Discussion

**Figure 1:**
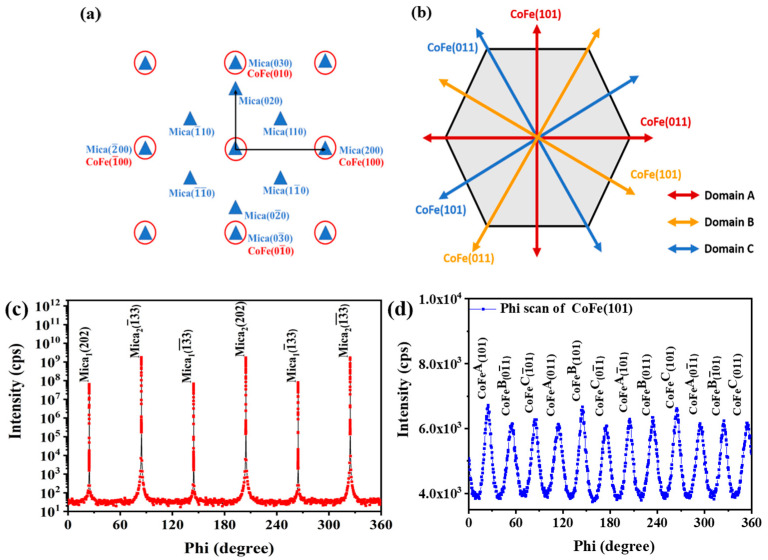
Schematic diagram of (**a**) reciprocal lattice of mica(001) and CoFe(001), (**b**) the reciprocal space of the CoFe(h k 1) (l = 1) plane and the arrangement of three CoFe crystal domains at the CoFe/mica interface, (**c**) azimuthal scan of mica{202}, and (**d**) CoFe{101} for CoFe thin films.

The thin films were grown at different growth temperatures ranging from room temperature to 500 °C. However, the XRD results for the CoFe films show a degradation in the peak intensity at different growth temperatures above 400 °C. A similar phenomenon has been studied previously, suggesting that annealing at higher temperatures results in the desorption of K from mica, consequently affecting and degrading the properties of the mica [31]. Therefore, in this work, we report only the measurements performed at a growth temperature of 400 °C. Figure 1a shows the reciprocal lattices of mica(001) and CoFe(001), while Figure 1b depicts the schematic representation of the arrangement of three domains in CoFe/mica along the azimuthal direction. Figure 1c displays the azimuthal scan, confirming the epitaxial relation existing between CoFe{101} and mica{202}. This relation exhibits a four-fold symmetry in each domain, forming three domains with each domain rotated by 120°. Since CoFe{101} and CoFe{011} are perpendicular to each other, a diffraction peak occurs after 30°, resulting in twelve diffraction peaks. There are twelve diffraction peaks from CoFe{101}, and six of them are at the same phi angle position with mica{202}. This indicates that CoFe(100) grows along the a-axis of mica. Since CoFe{101} diffraction signals are detected at chi = 45°, for the body center cubic structure of CoFe, it shows that the preferred orientation of CoFe is along the (002) direction on mica. Mica possesses a monoclinic structure with lattice parameters as follows: a = 5.308 Å, b = 9.183 Å, c = 10.141 Å, α = 90°, β = 100.07°, and γ = 90°. Whereas the lattice parameters of BCC CoFe are a = b = c = 2.855 Å and α = β = γ = 90°. Despite a 7.573% lattice mismatch between CoFe(100) and the a-axis of mica, crystalline CoFe films can be epitaxially grown on a mica substrate. These results suggest that the crystallography orientation of CoFe films on mica is CoFe(001)[100]//mica(001)[100] in three symmetric domains with each one rotating by 120°. Table 1 compares the lattice parameters of CoFe obtained from this work with the previously reported studies.

**Figure 2:**
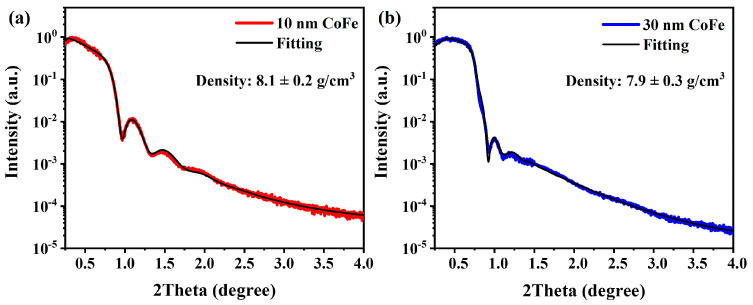
X-ray reflectivity curves for (**a**) 10 nm and (**b**) 30 nm CoFe/mica.

To determine the thickness of the deposited CoFe films, XRR measurements were utilized. Figure 2a and Figure 2b showcase the experimental results and corresponding fitting outcomes for films deposited for 10 min and 30 min, respectively. Based on the fitting results, we observed that the thickness of the thin films after 10 min and 30 min was 10 nm and 30 nm, respectively. This suggests that the deposition rate of our CoFe films is 1 nm/min. Furthermore, to achieve a thickness of 50 nm, the samples were deposited for a period of 50 min. The determined thickness of the CoFe thin film exhibits remarkable precision, with an uncertainty of about ±0.2 nm. This highly accurate thickness measurement becomes an important input parameter for Equation (2), enabling a reliable estimation of strain.

**Figure 3:**
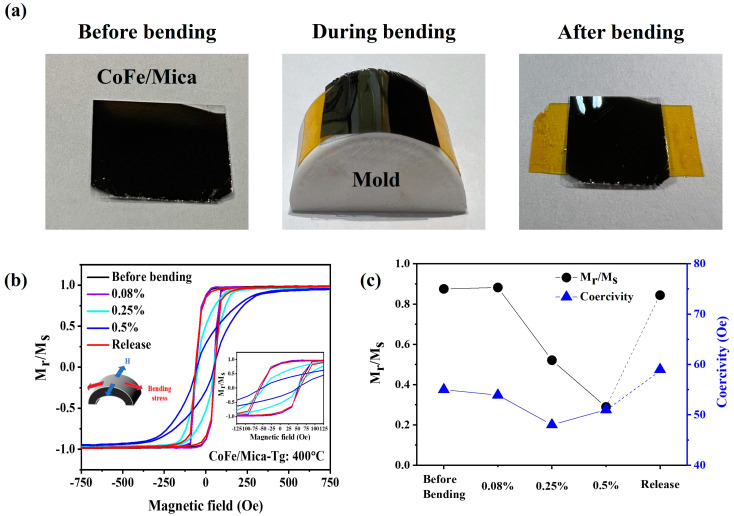
(**a**) Top and side view of CoFe/mica films before bending, during bending (0.25% strain), and after bending. (**b**) Hysteresis loops (in-plane) of CoFe/mica films measured under various tensile strains, with an inset showing the enlarged view of the magnetic field ranging from −125 Oe to 125 Oe. (**c**) Plot of squareness and H_c_ as a function of applied strain obtained from (**b**).

Figure 3a shows the top and side view of the CoFe/mica films before bending, during bending, and after bending. Figure 3b shows the hysteresis loops of CoFe/mica films sputtered at 400 °C, taken in an in-plane field reversal. The inset shows the magnetic field ranging from −125 Oe to 125 Oe. Based on the Villari effect, the remanence of a ferromagnet is enhanced when it features a positive magnetostriction constant together with tensile stress [39].

After harvesting the information regarding the microstructure and magnetic properties of CoFe/mica, the focus turns to the AMR response in CoFe/mica under various strain treatments. The strain was calculated using the radius of the curvature of the mold, and at 0.5% strain, the radius of curvature reaches 0.5 cm. Our observations revealed that beyond 0.5% strain, scanning electron microscopy analysis detected cracks on the CoFe surface. These cracks, once formed, resulted in irreversible changes in resistance, leading to an increase in resistance levels. Therefore, our study focused specifically on strains only up to 0.5%. Five strain states were intentionally set by mounting CoFe/mica on molds with various curvatures for strain-free, 0.08% tensile strain, 0.25% tensile strain, 0.5% tensile strain, and relaxation state. The applied strain in the samples was calculated using the following equation [40]:

(2) $$S=(\frac{t_{f}+t_{s}}{2R})(\frac{1+2\eta +\chi \eta^{2}}{\left(1+\eta )(1+\chi \eta \right)})$$

where S is strain, $\eta =\frac{t_{f}}{t_{s}},\chi =\frac{Y_{f}}{Y_{s}}$, and $t_{f}$ and $t_{s}$ are the thickness of thin film and substrate, respectively. *R* is the radius of the bending curvature. $Y_{f}$ and $Y_{s}$ are Young’s modulus of thin film and substrate, respectively.

Due to the positive Poisson ratio, there is a relative compressive stress orthogonal to the tensile stress along the bending direction. According to the Villari effect [41,42], magnetization tends to align along the tensile direction. From the M–H curves of CoFe/mica as shown in Figure 3c, two important signatures can be observed. Firstly, both squareness and coercivity of CoFe/mica are reduced with increasing strain. The coercivity values of CoFe thin films under different applied strain conditions are as follows: 55 Oe (before bending), 54 Oe (0.08% strain), 48 Oe (0.25% strain), 51 Oe (0.5% strain), and 59 Oe (release). Because the M–H curves were taken transversely to the bending direction as shown in the experimental geometry, the drops on both squareness and coercivity may suggest the opposite evolutions on the M–H curves taken along the bending direction, revealing a strong correlation between squareness/H_c_ and strain. In addition, increasing strain can disrupt the alignment of magnetic grains, potentially reducing the ratio of M_r_/M_s_ at 0.5% strain. On the other hand, H_c_ is influenced by various factors, including dislocations, and elongated grain sizes due to slip plane alterations during strain application. Consequently, establishing a direction correlation between M_r_ and H_c_ might not be straightforward due to these multifaceted influences. We note that this experimental geometry is to ensure that the magnetizations in the curved film are geometrically identical in the film to avoid any uniformity issue. In addition, both squareness and coercivity of CoFe/mica can return to their initial states as strain-free after releasing the strain, confirming the remarkable reversibility of the CoFe/mica films.

**Figure 4:**
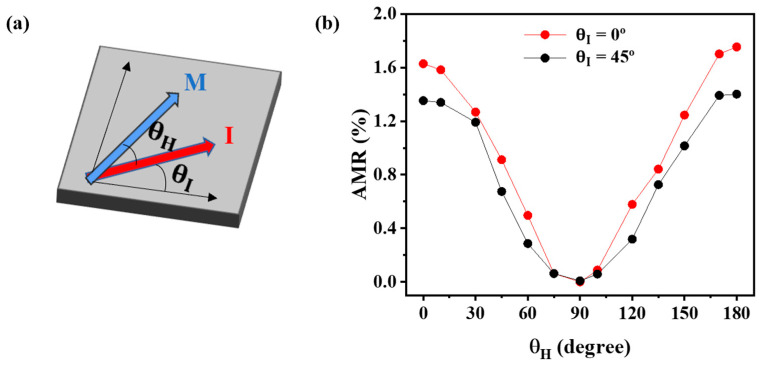
(**a**) Schematic representation of the magnetoresistance of epitaxial CoFe films and (**b**) on flexible mica substrate for a current along $\theta_{I}$ = 0° and $\theta_{I}$ = 45°.

Additionally, an angle-dependent resistance measurement was performed for the CoFe/mica as a representative to verify the correlation between the magnetism changes and the AMR effect. Figure 4a shows the experimental geometry of the angle-dependent resistance measurement in which a $\theta_{H}$ is defined by the angle between the magnetic field and applied current, which ranges from 0° to 180° due to the uniaxial characteristic of AMR and $\theta_{I}$ defined as the in-plane direction of the CoFe(100) direction at 0° in the four-fold symmetry epitaxial CoFe thin film.

**Figure 5:**
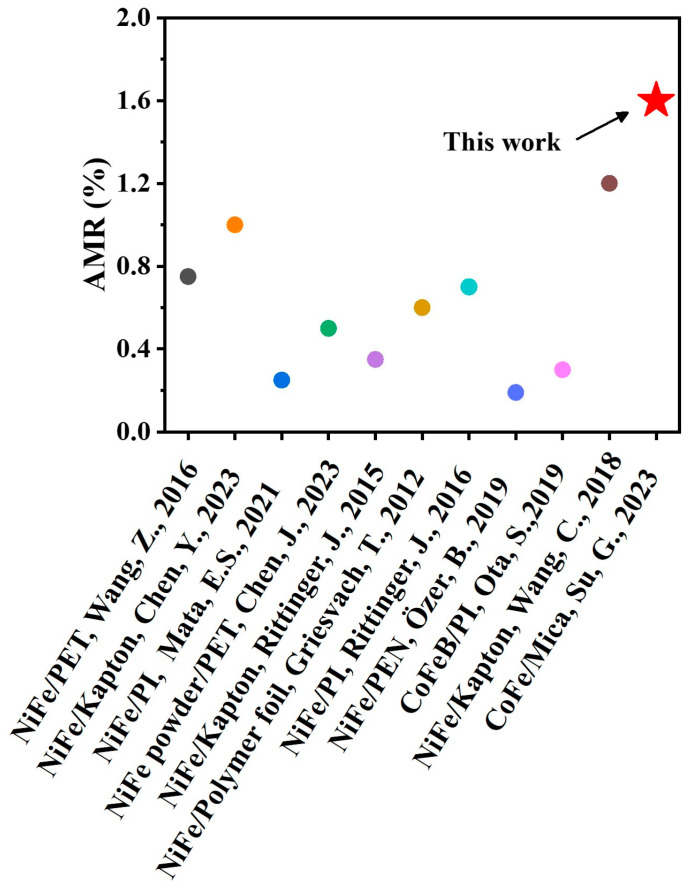
Plot summarizing the values of an AMR ratio in ferromagnetic thin films on various flexible substrates together with this present work [20,21,22,43,44,45,46,47,48,49].

Furthermore, we investigated the magnetoresistance of epitaxial CoFe films on a flexible mica substrate grown at 400 °C along different crystal axes, with the angle $\theta_{H}$ varying from 0° to 180°. The angular-dependent magnetoresistance of the epitaxial CoFe films on the mica substrate is weak, as shown in Figure 4b. The measured AMR ratio is 1.6% for $\theta_{I}$ = 0°, whereas it is around 1.4% for $\theta_{I}$ = 45°. This weak anisotropic AMR effect in epitaxial CoFe films on the mica substrate could be due to the three domains shown in the epitaxial condition for CoFe films on mica and poor orientation alignment between CoFe and mica (see the azimuthal scan in Figure 1c,d). It can be seen from Figure 5 that the AMR ratio in CoFe films on mica exhibited the highest AMR ratio when compared with the other ferromagnetic thin films grown over various flexible substrates. Table 2 provides the comparison of different permalloy and CoFe films grown on various substrates, their deposition methods, and their corresponding AMR ratios.

**Figure 6:**
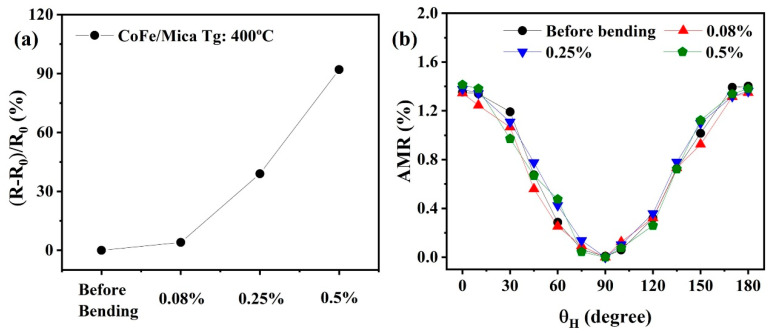
(**a**) Change in the resistance of CoFe thin films with respect to different bending conditions, and (**b**) AMR ratio of CoFe thin films on a flexible mica substrate with varying angles $\theta_{H}$ under different tensile strains at $\theta_{I}$ = 45°.

Furthermore, to confirm the feasibility of using CoFe thin films on mica as a wearable AMR sensor, it is crucial to study the AMR performance under different bending states, with the angle $\theta_{H}$ varying from 0° to 180°. Figure 6a displays the resistance change of CoFe/mica grown with various bending stresses where the resistance was taken along the bending direction. This change is primarily due to thin film cracks or dislocations occurring in a perpendicular direction to the tensile direction, resulting in more electron jumps or scattering. As a result, the resistance of CoFe films increases during the bending process. Figure 6b displays the AMR effect of CoFe films under various tensile strain conditions, and the change in the AMR ratio is close to 1.4% under different bending states at $\theta_{I}$ = 45°. Notably, even during bending, the flexible CoFe films exhibit a stable AMR response of approximately 1.4%, and the presence of thin film cracks and dislocations or even plastic deformations does not affect their performance as a magnetic sensor.

**Figure 7:**
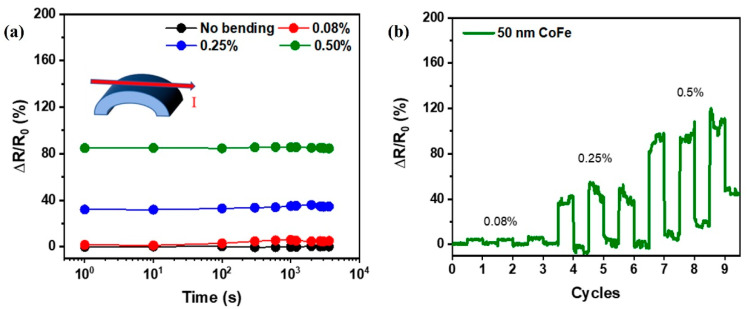
(**a**) Resistance testing of 50 nm CoFe/mica under cyclic stress with respect to various bending conditions. (**b**) The time-dependent resistance measurement results of 50 nm CoFe/mica under strain.

To further demonstrate the feasibility of CoFe/mica as a flexible device, we conducted tests, such as time-dependent resistance hysteresis and strain-stress cycling. These tests confirm the film’s stability during prolonged bending time and repeated strain-stress cycles. In Figure 7a, the resistance behavior of a 50 nm CoFe/mica film under various strains over time is depicted. The results exhibit consistent performance within the error range, indicating its suitability for flexible applications. In cyclic resistance change tests, an ideal AMR sensor should reliably return to its original value after strain release. Figure 7b illustrates resistance variations at 0.08%, 0.25%, and 0.5% bending strain over three cycles, each lasting 200 s. These data indicate consistent resistance changes for strains below 0.25%. However, when strains exceed 0.5%, the resistance change intensifies and does not fully revert back to the initial value. This behavior might be attributed to CoFe film creep during high-strain bending. While the sensor demonstrates stability at lower strains, further investigation is needed to address the increased resistance change under higher strains. Potential adjustments might be required to mitigate the effects of CoFe film creep. This thorough exploration will ensure the reliability and applicability of CoFe/mica in flexible devices, providing insights into its behavior under diverse conditions and helping enhance its performance for various applications. Overall, this study suggests that epitaxially grown CoFe thin films on a flexible mica substrate at T_g_ = 400 °C have the potential to be utilized as a wearable magnetoresistance sensor independent of the stress applied.

## 4. Conclusions

This work demonstrates a comprehensive analysis of the crystal structure, epitaxial relationship, and magnetic properties of CoFe films deposited onto a flexible mica substrate. Notably, this study identifies the formation of three CoFe (002) domains exhibiting a distinctive four-fold symmetry when deposited on mica. Through controlled applications of tensile strain via bending stress, we tuned the magnetic properties of CoFe films on the flexible mica substrate. Moreover, our investigations revealed the reversible nature of the magnetic characteristics exhibited by CoFe films on the flexible mica substrate upon the release of strain. Despite the weak dependence on the crystalline orientation, the AMR property in the epitaxial CoFe films on the flexible mica substrate demonstrated consistent behavior, maintaining a stable AMR ratio of 1.4% at $\theta_{I}$ = 45° across various bending states. Overall, this study not only highlights the potential of CoFe films on mica substrates for applications in wearable technology but also signifies their viability as key components in the development of magnetoresistance AMR sensors, offering both tunability and reliability in diverse bending conditions.

## Figures and Tables

**Table 1:** Comparison of lattice parameters of deposited CoFe in this work and the previously reported works.

Lattice Constant of CoFe	Sample Type	Reference
2.855 Å (Experimental)	CoFe powder	[32]
2.855 Å (Experimental)	CoFe powder	[33]
2.857 Å (Experimental)	CoFe powder	[34]
2.857 Å (Calculated)	Bulk CoFe	[35]
2.858 Å (Experimental)	Thin film—CoFe/n-Si	[36]
2.863 Å (Experimental)	Thin film—CoFe/p-Si	[37]
2.870 Å (Experimental)	Thin film—CoFe/MgO	[38]
2.855 Å (Experimental)	Thin film—CoFe/mica	This work

**Table 2:** Comparison of AMR ratio values of various permalloy and CoFe films deposited on various flexible substrates.

Material	Method of Preparation	Substrate Used	Thickness	AMR Ratio (%)	Ref.
Ni_0.81_Fe_0.19_	DC magnetron sputtering	Polyethylene terephthalate	30 nm	0.75	[20]
Ni_0.81_Fe_0.19_	DC magnetron sputtering	Kapton	10 nm	1	[21]
Ni_0.81_Fe_0.19_	DC magnetron sputtering	Mylar foil	100 nm	0.25	[22]
Ni_0.80_Fe_0.20_	Ion beam sputtering	Polyimide	60 nm	0.5	[43]
Ni_0.81_Fe_0.19_	RF sputtering	Kapton	100 nm	0.34	[44]
Ni_0.81_Fe_0.19_	Sputtering	Polymer foil	50 nm	0.6	[45]
Ni_0.81_Fe_0.19_	RF sputtering	Polyimide	100 nm	0.7	[46]
NiFe	Magnetron sputtering	Kapton	10 nm	0.19	[47]
Co_20_Fe_60_B_20_	Sputtering	Polyimide	2.7 nm	0.2	[48]
NiFe	Sputtering	Kapton	NA	1.2	[49]
Co_50_Fe_50_	DC magnetron sputtering	Mica	50 nm	1.6	This work

## Data Availability

The data presented in this study are available on request from the corresponding author.
